# Sex differences in the combined effect of diabetes and frailty on all-cause mortality in community-dwelling older adults

**DOI:** 10.3389/fendo.2025.1670278

**Published:** 2025-10-23

**Authors:** Nina Mielke, Muhammad Helmi Barghouth, Alice Schneider, Damiano Ferrari, Janine Kaiser, Natalie Ebert, Elke Schaeffner

**Affiliations:** ^1^ Charité – Universitätsmedizin Berlin, corporate member of Freie Universität Berlin and Humboldt Universität zu Berlin, Institute of Public Health, Berlin, Germany; ^2^ Charité – Universitätsmedizin Berlin, corporate member of Freie Universität Berlin and Humboldt Universität zu Berlin, Institute of Biometry and Clinical Epidemiology, Berlin, Germany

**Keywords:** frailty, diabetes, mortality, older adults, cohort study, sex-specific, sex-frailty paradox, healthy aging

## Abstract

**Introduction:**

Diabetes and frailty are common in older adults and independently associated with all-cause mortality. Sex-stratified analyses indicate that the mortality risk associated with frailty is higher in men, whereas that associated with diabetes is higher in women. This study investigates sex differences in the combined effect of diabetes and frailty on mortality.

**Methods:**

The Berlin Initiative (cohort) study assessed frailty and diabetes at the third follow-up visit. Participants (women and men ≥75 years) were categorized by frailty and diabetes status and followed-up until death or dataset closure (March 2023; median follow-up 6.0 years). Sex-stratified Cox regressions estimated hazard ratios (HRs) and 95% confidence intervals (CI) for all-cause mortality. Interaction between frailty and diabetes on mortality was analysed.

**Results:**

Of 1143 participants (mean age 84 years; 55% women), baseline characteristics were similar between sexes, with fewer women having a partner (33% vs. 70%) or cardiovascular disease (67% vs. 77%). Mortality risk increased from non-frail individuals with diabetes (HR, 95% CI: women 1.25, 0.76-2.06 vs. men 1.56, 1.08-2.24) to those with frailty alone (HR 95% CI: women 1.81, 1.26- 2.59 vs. men 2.52, 1.78-3.57) and was highest among those with both conditions (HR 95% CI: women 3.39, 2.19-5.24; men 3.42, 2.28-5.14). An indication of additive interaction between frailty and diabetes on mortality was only found in women (RERI 1.32, 95% CI 0-2.65).

**Conclusions:**

Diabetes and frailty increase mortality risk in both older women and men, with an additive interaction in women. These findings support sex-specific risk stratification and emphasize the need for mechanistic research to inform targeted interventions.

## 1 Introduction

Diabetes mellitus and frailty are major health concerns in ageing societies (1). Although the overall mortality rate in individuals with diabetes type 2 has decreased, due to the increasing emphasis on integrated care of patients with chronic diseases, improved patient education in disease management and advances in clinical decision making, it remains higher compared to the general population (2, 3). Recent therapeutic advances, including sodium-glucose cotransporter-2 inhibitors (SGLT2i) and glucagon-like peptide-1 receptor agonists (GLP-1 RA), have contributed to improved clinical outcomes and reduction in mortality (4, 5). The global age-standardised diabetes prevalence is expected to increase by 59.7% by 2050, reaching an estimated 9.8% of the population (6).

In older adults aged 65–95 years, the global diabetes prevalence is above 20% being highest in the age group of 75–79 years with 24.4% (6). Older adults with diabetes are at increased risk of institutionalization, accelerated muscle loss, more functional disability and multimorbidity as well as mortality compared to those without (7). In addition to age, the excess mortality risk in individuals with diabetes varies depending on disease duration and sex (8). The excess mortality risk in individuals with diabetes is higher in women compared to men within the same age group (8–10). Sex differences in mortality risk decrease with increasing age at diagnosis until no difference is observed in older adults diagnosed at the age of 70 or older (9). Furthermore, a systematic review showed that individuals diagnosed with diabetes at age 70 or older do not have an increased mortality risk at all compared to those without diabetes (11).

Frailty may partly explain the excess mortality risk in individuals with diabetes (12). A recent meta-analysis showed that about half of the older individuals with diabetes are frail (13). Frailty refers to a biological syndrome characterized by decreased reserve across multiple physiological systems and an increased vulnerability and susceptibility to adverse events such as hospitalisation, falls, admission to long-term care and mortality (14–16). Regarding sex differences in frailty, it has been shown that the mortality risk in frail older men is greater compared to frail older women although the prevalence of frailty is higher in women (17). Thus, this ‘sex-frailty paradox’ represents another conceptualization of the male-female health-survival paradox (18).

Despite the knowledge of sex differences in mortality from both diabetes and frailty, most studies analyzing diabetes and frailty with respect to mortality risk did not address these differences (19–28). To our knowledge, only one study addressed sex differences of diabetes and frailty on risk of mortality (29). Cacciatore et al. analysed data from Italy, showing that men had a higher mortality rate than women across each subcategory of combined frailty and diabetes status over a 12-year follow-up period and found no significant multiplicative interaction between frailty and sex on mortality risk (29). However, a systematic review and meta-analysis on frailty measurement in individuals with diabetes found that their frailty measure had not been used in any other study (30).

Using the frequently applied frailty phenotype (14), we aim to analyse sex differences in the association of a combination of diabetes and frailty on mortality risk using data on old and very old adults from the Berlin Initiative Study (BIS). The addressed research questions are: (1) Are there sex differences in the mortality risk in community-dwelling older adults with and without diabetes depending on their frailty status? and (2) is there an interaction of diabetes and frailty with regard to mortality risk on the multiplicative or additive scale in men, women or both?

## 2 Methods

### 2.1 Study population

The Berlin Initiative Study (BIS) is a cohort study of 2069 community-dwelling older adults with enrolment between November 2009 and July 2011. The inclusion criteria for the BIS were a minimum age of 70 years and membership of the statutory health insurance fund AOK Nordost – Die Gesundheitskasse (AOK). Exclusion criteria at BIS baseline were nursing cases, dialysis patients, or kidney transplant recipients (31). Primary data were collected at 2-year intervals by face-to-face interviews augmented by anthropometric measurements, laboratory measures and geriatric assessments after written informed consent was obtained. These data were then complemented by AOK health claims data linked on person-level. Frailty assessment was implemented at the third follow-up visit (2016-2017) which constitutes the baseline for this study. All participants with a valid frailty assessment and diabetes status were included resulting in a study population of 1143 participants (**Supplementary Figure 1**). The study was approved by the ethics committee, Charité – Universitätsmedizin Berlin, Germany (EA2/009/08) and is in accordance with the 1964 Helsinki declaration and its later amendments.

### 2.2 Exposures: diabetes and frailty

Diabetes was defined as a glycated hemoglobin A1c (HbA1c) level ≥6.5% or intake of any antidiabetic drug (anatomical therapeutic chemical (ATC) classification system: A10B or A10A). Medication was assessed by medically trained staff based on the packages, lists, and the participants’ self-report, and entered into a standardized computer-based questionnaire linked to a drug database to provide additional information such as ATC codes (32). Of the 296 participants with diabetes, 21% (n=63) did not report taking antidiabetic drugs and thus were classified based on an elevated HbA1c. To address the potential for misclassification, diabetes status was verified using AOK data, where 97.3% of the 296 individuals were assigned the ICD-10 code E11 for type 2 diabetes (**Supplementary A**).

Frailty was assessed by applying the five Fried criteria, of which shrinking, exhaustion and weakness were assessed without modification (14). Low physical activity was assessed as engaging in physical activity (e.g., brisk walking, that exceeds 30 minutes) less than once a week and slowness if 15 seconds or more were needed to perform the Timed Up and Go test (33, 34). Participants who met more than three of the five criteria were defined as frail and all other participants were defined as non-frail.

Based on both their diabetes and frailty status, participants were categorized into four categories: (1) neither frail nor diabetic, (2) diabetic but not frail, (3) not diabetic but frail, and (4) both diabetic and frail.

### 2.3 Outcome: all-cause mortality

The mortality date from all causes was determined from AOK health claims data (available in all cases) and validated by death certificates.

### 2.4 Covariable assessment

Primary data assessed during BIS study visits included information on demographics, lifestyle variables and morbidity collected using a standardized computer-based questionnaire. Anthropometric and geriatric assessments as well as laboratory tests were also carried out (**Supplementary B**). The primary study data were complemented by AOK health claims data linked on person-level, providing e.g., morbidities coded according to the 10th Revision of the International Statistical Classification of Diseases and Related Health Problems, (ICD-10) and information on participants who could no longer be followed-up.

The following covariates were derived from BIS data: age, sex, the short version of the CASMIN (Comparative Analysis of Social Mobility in Industrial Nations) classification of education (35), partner status (yes/no), body mass index (BMI) in kg/m^2^, and smoking status (never, ever). The following additional variables such as morbidities and laboratory parameters were used: arterial hypertension defined as regular intake of antihypertensive medications and chronic kidney disease (CKD) defined as either a glomerular filtration rate estimated based on the BIS2 equation (eGFR) of <60 ml/min per 1.73 m^2^ (36) or albuminuria defined as an albumin-creatinine ratio (ACR) ≥30 mg/g. C-reactive protein (CRP; mg/l) and total cholesterol (mg/dl) were analyzed using blood serum. Age of diabetes onset was calculated based on self-report and later dichotomized in <70 and ≥70 years. Cardiovascular disease (CVD) is a composite variable ascertained by a combination of self-reported BIS data validated by ICD-10 coded medical reports and AOK claims data defined as either having a history of stroke (ICD-10: I61, I63, I64, I69.1-4), history of myocardial infarction (I21, I22, I23, I25.2), congestive heart failure (ICD-10: I11.0, I13.0, I13.2, I25, I50) or peripheral vascular disease (PVD: self-reported intermittent claudication or angiography, bypass or amputation due to PVD or ICD-10: I70.2, I73.1, I73.9). The Charlson Comorbidity Index (CCI) (37) was used as a measure of morbidity and was compiled from the AOK health claims data.

### 2.5 Statistical analyses

The characteristics of participants at study baseline are described stratified by sex with means and standard deviations (SD) for continuous variables or as medians and interquartile ranges (IQR) depending on the distribution. Categorical variables are presented in absolute and relative frequencies. All participants were followed from study baseline until death or the end of the study period (March 31st, 2023), whichever occurred first.

The Kaplan-Meier method was applied to estimate survival probabilities of combined diabetes and frailty categories with mortality stratified by sex. A log-rank test was used to test whether the difference between survival times of the groups was statistically different. The association of combined diabetes/frailty categories with all-cause mortality was further analyzed using Cox proportional hazard models to estimate crude and adjusted hazard ratios (HR) with 95% confidence intervals (CI) in women and men. To address confounding, a knowledge-based pre-specified adjustment set included: age, smoking, partner status, CASMIN, CRP, BMI, hypertension, total cholesterol, CKD and CVD. Due to missing data for the covariables (n=61, **Supplementary Figure 1**), the Cox proportional hazard models were computed for 1082 participants.

Interaction of diabetes and frailty on the outcome all-cause mortality was investigated on multiplicative (combined effect of frailty and diabetes on mortality exceeds the product of their individual effects, indicating relative risk synergy that my reflect underlying biological interaction) and additive (combined effect of frailty and diabetes on mortality exceeds the sum of their individual effects, reflecting the absolute excess risk important for public health) scales using (1) dichotomized diabetes and frailty variables with an interaction term in the models and (2) the approach suggested by Knol and VanderWeele (38) using the InteractionR package (39). Model-adjusted risks with corresponding 95% CI and measures of interaction on both multiplicative (Ratio of HRs) and additive (Relative Excess Risk due to Interaction, RERI) scales were computed (40).

In a sensitivity analysis, the age of diabetes onset was added to the adjustment set as a proxy for disease duration. The dataset was stratified by diabetes status and the models were repeated. The adjustment set in the diabetes stratum included: age, sex, smoking, partner status, CASMIN, CRP, BMI, hypertension, total cholesterol, CKD, CVD and age of diabetes onset.

To facilitate comparison with studies that did not stratify by sex, all analyses were additionally performed in the total study population. In these models, the adjustment sets additionally included sex. (**Supplementary C**).

For all analyses, two-sided P ≤ 0.05 was considered statistically significant. All statistical analyses were conducted with R (Version 4.1.1; R Foundation for Statistical Computing, Vienna, Austria) and reporting of results was performed according to the Strengthening the Reporting of Observational Studies in Epidemiology (STROBE) statement (**Supplementary D**).

## 3 Results

### 3.1 Sex-differences in main characteristics of the study population

Overall, the mean age was 84 years for both women and men (**Table 1**). Of the 1143 participants, 629 (55%) were women. They had less often diabetes (23.4%) compared to men (29.0%). Of 149 men with diabetes, 58 (38.9%) were frail, while out of 147 women, almost half (46.9%) were frail. The prevalence of arterial hypertension was high in both groups, reported by 84% of women and 82% of men. CKD was also common, present in 77% of women and 79% of men. A substantially higher proportion of men reported having a partner (70%) compared to women (33%). The prevalence of ever smoking was more than twice as high in men (68%) than in women (28%). Additionally, the prevalence of cardiovascular disease (CVD) was more frequent among men (77%) than women (67%).

**Table 1:** Main characteristics of study population stratified by sex.

	Total (n = 1143)	Women (n = 629)	Men (n = 514)
Age (Years), mean (SD)	84.3 (5.6)	84.0 (5.6)	84.3 (5.7)
CASMIN, n (%)			
Low	677 (59)	412 (66)	265 (52)
Intermediate	234 (20)	147 (23)	87 (17)
High	227 (20)	69 (11)	158 (31)
Missing	5 (0.4)	1 (0.2)	4 (0.8)
Partner Status, n (%)			
Yes	569 (50)	208 (33)	361 (70)
Missing	4 (0.4)	3 (0.5)	1 (0.2)
Body Mass Index (kg/m^2^), n (%)			
< 22	112 (10)	78 (12)	34 (7)
≥ 22 - < 30	763 (67)	392 (62)	371 (72)
≥ 30	255 (22)	154 (24)	101 (20)
Missing	13 (1.1)	5 (0.8)	8 (1.6)
Smoking, n (%)			
Never	615 (54)	453 (72)	162 (32)
Ever	525 (46)	174 (28)	351 (68)
Missing	3 (0.3)	2 (0.3)	1 (0.2)
Total Cholesterol (mg/dl)			
Mean (SD)	200 (48)	220 (48)	190 (41)
Missing (%)	20 (1.7)	15 (2.4)	5 (1.0)
C-reactive Protein (mg/l)			
Median (IQR)	1.7 (0.9, 3.6)	1.7 (0.9, 3.6)	1.6 (0.8, 3.5)
Missing (%)	23 (2.0%)	18 (2.9)	5 (1.0)
Charlson Comorbidity Index			
Median (IQR)	6 (3, 8)	5 (3, 7)	6 (4, 9)
Missing	15 (1.3%)	9 (1.4)	6 (1.2)
Treated Arterial Hypertension, n (%)			
Yes	947 (83)	528 (84)	419 (82)
Missing	1 (0.1)	1 (0.2)	
Cardiovascular Disease, n (%)			
Yes	817 (71)	422 (67)	395 (77)
Missing	11 (1.0)	6 (1.0)	5 (1.0)
Chronic Kidney Disease, n (%)			
Yes	890 (78)	485 (77)	405 (79)
Missing	30 (2.6)	24 (3.8)	6 (1.2)
Diabetes/Frailty combination, n (%)			
No diabetes/Non-Frail	586 (51)	318 (51)	268 (52)
Diabetes/Non-Frail	169 (15)	78 (12)	91 (18)
No diabetes/Frail	261 (23)	164 (26)	97 (19)
Diabetes/Frail	127 (11)	69 (11)	58 (11)
Age of Diabetes Onset, n (%)			
<70 years	116 (10)	50 (8)	66 (13)
>=70 years	165 (14)	90 (14)	75 (15)
No DM	847 (74)	482 (77)	365 (71)
Unknown	15 (1)	7 (1)	8 (2)

CASMIN Comparative Analysis of Social Mobility in Industrial Nations, BMI body mass index, CKD chronic kidney disease defined as either glomerular filtration rate of <= 60 ml/min per 1.73 m^2^ or ACR (albumin-creatinine ratio) >= 30 mg/g, hypertension defined as intake of any antihypertensive medication, cardiovascular disease (ever stroke, ever myocardial infarction, congestive heart failure or peripheral vascular disease).

### 3.2 Combined diabetes/frailty

The distribution of combined diabetes and frailty categories was similar between women and men. Most participants had neither diabetes nor frailty (women: 51%, men: 52%), with small differences in diabetic non-frail individuals (12% vs. 18%) and non-diabetic frail individuals (26% vs. 19%), while 11% of both sexes were diabetic and frail (**Table 1**).

Although the overall patterns of the main characteristics were comparable between sexes, some differences were found (**Supplementary Tables 1**, **2**). In individuals with either frailty or diabetes the levels of CRP differed. In non-frail diabetic women, CRP levels were higher compared to non-diabetic frail women (2.5 vs. 1.9 mg/L), whereas in men, CRP levels were higher in non-diabetic frail than in diabetic non-frail individuals (2.6 vs. 1.7 mg/L). CVD prevalence was higher with the presence of diabetes or frailty in both sexes. However, in frail women there was a difference between those with (91%) and without (77%) diabetes while in men the prevalence of CVD was high independent of the diabetes status (without diabetes 96%, with diabetes 91%).

### 3.3 Sex differences on the effect of Diabetes/Frailty combination on survival

**Figure 1:**
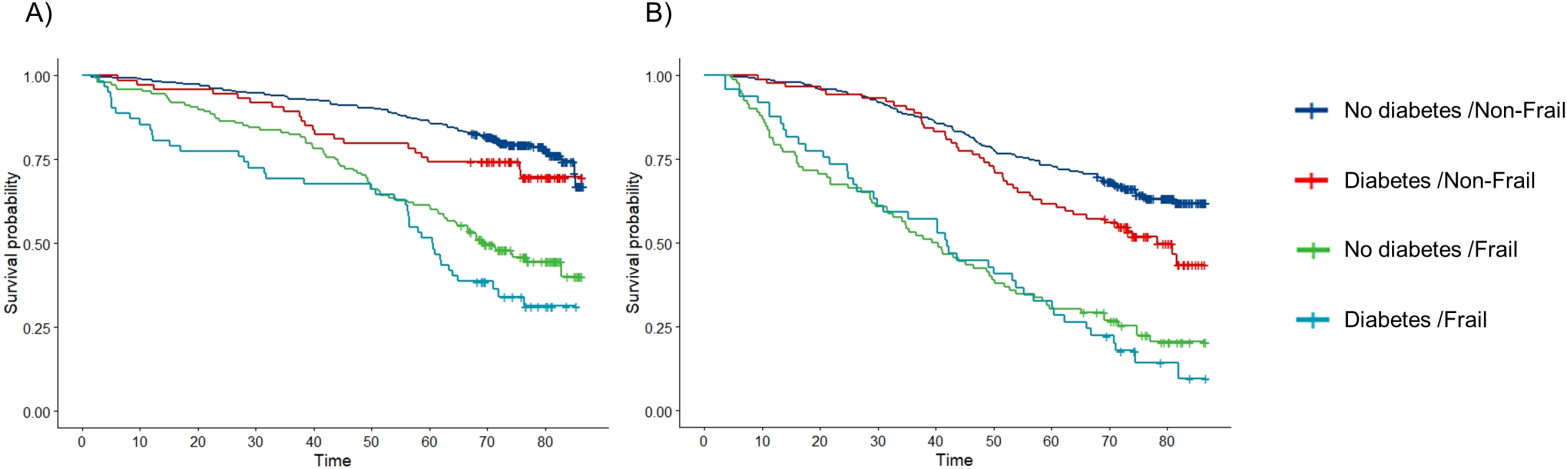
Kaplan-Meier curves of the survival probabilities over time (months) by combined diabetes/frailty categories in **(A)** women and **(B)** men.

During the median (IQR) follow-up of 6.0 (4.0-6.6) years (women 6.1 (6.0-6.7) and men 5.9 (3.3-6.4)), out of 629 women 224 (36%) died and out of 514 men 269 (52%) died. The cumulative survival curves stratified by sex are displayed in **Figure 1**. The median survival time of men was lower compared to women (S**upplementary Tables 3**, **4**). In men, the survival seemed to be dependent on the frailty status but not on diabetes while in women, the Kaplan-Meier curves showed a decreasing survival with frailty, diabetes and both. The log-rank test showed a statistically significant difference in survival times between the groups. The median survival time for frail men independent of diabetes was about 40 months compared to 78 months in non-frail men with diabetes. In women, the median survival time was longer and differently dependent on frailty and diabetes status. The median survival time of frail women with diabetes was 60 months compared to 70 months for frail women without diabetes.

In the sex stratified multivariable-adjusted Cox models, the HRs for the combined categories of diabetes and frailty on all-cause mortality were higher in men than women (**Tables 2**, **3**). Among non-frail individuals with diabetes, the HR (95%-CI) for all-cause mortality was 1.25 (0.76-2.06) in women and 1.56 (1.08-2.24) in men. In frail individuals without diabetes, HRs were 1.81 (1.26-2.59) for women and 2.52 (1.78-3.57) for men. For those with both frailty and diabetes, mortality risk was similarly elevated in both sexes with 3.39 (2.19-5.24) in women and 3.42 (2.28-5.14) in men compared to non-frail non-diabetic individuals. In women, there was a notable indication of additive interaction between diabetes and frailty on mortality risk (RERI: 1.32 [0-2.65]), suggesting that their combined effect may exceed the sum of their individual effects.

**Table 2:** Interaction between diabetes and frailty on the risk of mortality in women.

		Number of participants	Deaths	Person years	HR (95% CI)
Combined categories					
No diabetes/Non-Frail		305	68	1808	Reference
Diabetes/Non-Frail		74	21	410	1.25 (0.76-2.06)
No diabetes/Frail		148	80	724	1.81 (1.26-2.59)
Diabetes/Frail		62	41	264	3.39 (2.19-5.24)
Stratified by Frailty					
Non-Frail	No Diabetes	305	68	1808	Reference
	Diabetes	74	21	410	1.25 (0.76-2.06)
Frail	No Diabetes	148	80	724	Reference
	Diabetes	62	41	264	1.87 (1.24-2.83)
Stratified by diabetes					
No Diabetes	Non-Frail	305	68	1808	Reference
	Frail	148	80	724	1.81 (1.26-2.59)
Diabetes	Non-Frail	74	21	410	Reference
	Frail	62	41	264	2.71 (1.57-4.66)
Ratio of HRs (95% CI)					1.50 (0.79-2.82)
RERI (95% CI)					1.32 (0-2.65)

RERI, Relative excess risk due to interaction; HR, Hazard ratio; 95% CI, 95% Confidence interval.

All HRs are adjusted for age, CASMIN, Partner Status, BMI, smoking, Total cholesterol, CRP, Hypertension, CVD and CKD.

**Table 3:** Interaction between diabetes and frailty on the risk of mortality in men.

		Number of participants	Deaths	Person years	HR (95% CI)
Combined categories					
No diabetes/Non-Frail		263	94	1451	Reference
Diabetes/Non-Frail		89	45	465	1.56 (1.08-2.24)
No diabetes/Frail		92	71	325	2.52 (1.78-3.57)
Diabetes/Frail		49	42	176	3.42 (2.28-5.14)
Stratified by Frailty					
Non-Frail	No Diabetes	263	94	1451	Reference
	Diabetes	89	45	465	1.56 (1.08-2.24)
Frail	No Diabetes	92	71	325	Reference
	Diabetes	49	42	176	1.36 (0.91-2.03)
Stratified by diabetes					
No Diabetes	Non-Frail	263	94	1451	Reference
	Frail	92	71	325	2.52 (1.78-3.57)
Diabetes	Non-Frail	89	45	465	Reference
	Frail	49	42	176	2.20 (1.42-3.42)
Ratio of HRs (95% CI)					0.87 (0.51-1.50)
RERI (95% CI)					0.35 (-0.99-1.69)

RERI: Relative excess risk due to interaction; HR: Hazard ratio; 95% CI: 95% Confidence interval

All HRs are adjusted for age, CASMIN, Partner Status, BMI, smoking, Total cholesterol, CRP, Hypertension, CVD and CKD

The sensitivity analysis showed consistent results, regardless of whether diabetes duration was considered (**Table 4**).

**Table 4:** Sensitivity analyses on the interaction between sex and frailty on the risk of mortality in individuals with diabetes addressing diabetes duration.

		Number of participants	Deaths	Person years	Model A HR (95% CI)	Model B including diabetes duration HR (95% CI)
Combined categories						
Men/Non-Frail		89	45	1916	Reference	Reference
Women/Non-Frail		74	21	2218	0.47 (0.26-0.86)	0.48 (0.26-0.87)
Men/Frail		49	42	501	2.05 (1.29-3.25)	1.99 (1.26-3.16)
Women/Frail		62	41	988	1.38 (0.81-2.35)	1.38 (0.82-2.34)
Stratified by Frailty						
Non-Frail	Men	89	45	1916	Reference	Reference
	Women	74	21	2218	0.47 (0.26-0.86)	0.48 (0.26-0.87)
Frail	Men	49	42	501	Reference	Reference
	Women	62	41	988	0.67 (0.40-1.12)	0.69 (0.41-1.16)
Stratified by sex						
Men	Non-Frail	89	45	1916	Reference	Reference
	Frail	49	42	501	2.05 (1.29-3.25)	1.99 (1.26-3.16)
Women	Non-Frail	74	21	2218	Reference	Reference
	Frail	62	41	988	2.92 (1.68-5.09)	2.89 (1.66-5.03)
Ratio of HRs (95% CI)					1.43 (0.72-2.84)	1.45 (0.73-2.89)
RERI (95% CI)					-0.14 (-1.04-0.76)	-0.09 (-0.98-0.80)

RERI, Relative excess risk due to interaction; HR, Hazard ratio; 95% CI, 95% Confidence interval.

Model A is adjusted for age, CASMIN, Partner Status, BMI, smoking, Total cholesterol, CRP, Hypertension, CVD and CKD.

Model B is adjusted for Model A and age of diabetes onset.

## 4 Discussion

Our study demonstrated that in both women and men, the HRs for mortality were higher in frail individuals, irrespective of diabetes status. Across diabetes and frailty categories, median survival was longer in women compared to men. Considering both conditions together, adjusted HRs for mortality increased progressively across categories: from non-frail individuals with diabetes to frail individuals with no diabetes to individuals with both compared to non-frail individuals without diabetes. Among individuals with both diabetes and frailty, HRs for mortality were similar in both sexes. In individuals that were either frail or diabetic the HRs for mortality were higher in men. This difference in mortality risk between women and men in the HRs for individuals with only one of the two conditions translates into a possible additive interaction of diabetes and frailty on mortality risk in women, but not in men.

The diabetes prevalence of 26% in this study is comparable to that of a study using the German Institute of Medical Documentation and Information (DIMDI) dataset which provides comprehensive routine data from German statutory health insurance funds (41). Comparing the sex-stratified results, the diabetes prevalence is higher in men compared to women, which is similar to our results. Regarding frailty, the prevalence within the age-strata is similar to that reported in a systematic review and meta-analysis of 62 countries worldwide (42). The sex-stratified frailty prevalence is only reported for all individuals 50 years and older and thus not comparable. In general, frailty prevalence is consistently higher in women than in men, a pattern that is also observed in our study.

Older individuals with diabetes have an increased risk of frailty compared to those without diabetes (25) and frailty is associated with increased risk for all-cause mortality in individuals with diabetes (30). The risk factors for diabetes become increasingly significant with age as physiological and lifestyle factors shift, including excess body weight, sedentary behaviour and dietary changes (7). These risk factors partly share common pathways for frailty which is the basis for analysing their interaction on the risk of mortality (1).

It has been established that frail individuals with diabetes have a higher mortality risk than non-frail individuals with diabetes (43). However, as the excess mortality risk associated with diabetes declines with age, its relevance in older adults remains uncertain. Therefore, in older adults, frailty and mortality risk should be assessed irrespective of diabetes status and potential interactions between frailty and diabetes should be evaluated.

To our knowledge, there are a few studies that examined the combined effect of frailty and diabetes on all-cause mortality in individuals with and without diabetes addressing a possible interaction (24, 25, 28). In one study from Japan, mortality risk seems to be driven mostly by frailty and not diabetes in individuals 65 years and older, although the risk of mortality is slightly higher in non-frail diabetic individuals (24). In the other study using data from China, the adjusted HRs for mortality increase with diabetes and frailty but not in individuals 80 years and older (25). In our study of individuals with a mean age of 84.3 years, the adjusted HRs for mortality increased with diabetes and frailty. A possible explanation for the differences in results compared to the Chinese study is that the authors adjusted only for age, sex, education and marital status, whereas our analyses included a more comprehensive adjustment set. Nevertheless, a similar pattern of survival probabilities across categories was observed in the Kaplan–Meier curves from our total study population and those from China, Japan, and Ireland, despite differences in median survival times between studies. (24, 25, 28).

In accordance with the study by Cacciatore et al. (29), the sex-stratified Kaplan-Meier curves indicate differences in mortality risk between men and women. Similar to our results, independent of diabetes status, frailty increased the risk of mortality in both men and women. We found that the pattern in women is similar to that of the total population but in men the mortality risk only seems to be modified by frailty. There is evidence indicating that diabetic men over the age of 65 years have no excess mortality compared to men of the same age without diabetes (44). Therefore, mortality risk is only changing through frailty and not through diabetes. Contrary to our results, the HRs were always higher in men. The difference in results may be explained by the age difference (our study population being on average 10 years older) and the usage of a different frailty assessment instrument. Interestingly, a recent systematic review and meta-analysis of 118 studies on frailty measurement in individuals with diabetes identified only the study by Cacciatore et al. (29) as having used their specific frailty instrument, whereas the frailty instrument applied in our study was used in 58% of the included studies (30).

Recognizing that sex differences have been shown in several underlying pathophysiological mechanisms common to diabetes and frailty on mortality including chronic inflammation, insulin resistance, oxidative stress and mitochondrial dysfunction and sarcopenia (1), we build upon the findings of previous studies by incorporating a sex-specific approach. In older adults no significant interaction between diabetes and frailty with regard to increased mortality risk has been shown so far (24, 29). Our results indicate that in individuals with either diabetes or frailty the mortality risk differs in men and women which could be due to sex-differences in combined pathways of frailty and diabetes and the risk of mortality.

Sex differences may, for example, involve chronic inflammation, as reflected by CRP levels, which can contribute differently to mortality (45). In this study, CRP distributions varied by exposure category and differed between women and men.

Elevated risks of endothelial dysfunction and vascular aging as well as insulin resistance and metabolic dysfunction can lead to elevated risks of CVD, CKD and mortality differently in women and men (46). While women are more prone to adiposity but preserve muscle mass, men experience greater loss of muscle mass and strength, resulting in more severe frailty and higher mortality risk (47). In our study population, the distribution of adiposity, as indicated by a BMI of ≥30kg/m^2^, varied across exposure categories between sexes. At the same time, CVD, further down on the pathway leading to mortality, was almost equally distributed across exposure categories in both women and men.

Sarcopenia and muscle wasting can contribute to frailty by reducing strength, increasing the risk of falls and accelerating functional decline. Additionally, they may exacerbate insulin resistance due to reduced glucose uptake in muscles, contributing to diabetes and increasing mortality risk through elevated risk of disability, fractures and hospitalization. The potential underlying mechanisms for the sex differences may include variations in muscle quality and in the regulation of muscle mass, such as differences in myostatin - a key factor in sex-specific patterns of sarcopenia and muscle wasting (48).

With regard to molecular differences, e.g. in relation to inflammatory processes, much more research is needed to better identify sex-specific differences as current studies have not placed much emphasis on sex differences. A recent review on the clinical aspects and molecular mechanisms of cardiac ageing and the sex-associated differences concluded that the lack of translation between basic science and clinical studies, along with the underrepresentation of women in these studies, has left this topic insufficiently researched yet (46).

Our study has several strengths including a well-phenotyped cohort of very old adults with comprehensive primary data complemented by secondary individual claims data and a median (IQR) follow-up of 6.0 (4.0-6.6) years. Secondly, through the combination of primary and secondary data the death status of all study participants was known even for those who were lost to follow-up. Thirdly, diabetes definition was based on a thorough assessment including a laboratory assessment and medication screening, with additional validation through identification of diabetes-specific ICD-10 codes in AOK claims data. Furthermore, we analysed interaction not only on the multiplicative but also on the additive scale to assess its public health importance. However, there are also some limitations we would like to address. We were not able to account for time-varying exposures. Information on e.g. diabetes was assessed at frailty baseline and not updated during the follow-up period. As a result, individuals who may have developed diabetes during the follow-up period could have been misclassified. But this potential misclassification would likely have led to an underestimation of the effect of diabetes and frailty on mortality. Secondly, the definition of diabetes used in this study did not account for disease duration. However, we used age of diabetes onset as a proxy for diabetes duration in the sensitivity analysis and found similar results to the primary analyses. This could be due to a survivor effect, where individuals with more severe forms of diabetes have died prior to study inclusion. However, our research question focuses on individuals who have reached an old age and whether there is an interaction between diabetes and frailty on mortality risk and whether this differs between sexes.

In conclusion, our findings demonstrate an additive interaction between diabetes and frailty on all-cause mortality in women, but not in men. The interaction probably reflects the comparatively lower mortality risk observed in women when only one condition is present, which amplifies the relative impact when both coexist. These sex-specific findings underline the complexity of multimorbidity in old age and the need to investigate the physiological mechanisms, ultimately guiding the development of targeted, sex-tailored interventions. Our findings affirm the importance of sex-stratified analyses in geriatric research and ensuring that sex differences are considered in clinical risk assessment and management for older adults.

## Data Availability

The data used in this study cannot be made available due to German data protection laws (Bundesdatenschutzgesetz). To facilitate the replication of results, the used data will be stored on a secure drive at Charité – Universitätsmedizin Berlin. Access to the raw data used in this study can only be provided to external parties under the conditions of a cooperation contract and can be accessed upon request, after written approval (bis@charite.de), if required.
