# Supplementary material for: Sex differences in the combined effect of diabetes and frailty on all-cause mortality in community-dwelling older adults
Source: Front Endocrinol (Lausanne). 2025 Oct 23;16:1670278. doi: 10.3389/fendo.2025.1670278 (PMC12588834; doi:10.3389/fendo.2025.1670278)
Supplement: Supplementary file 1 [file DataSheet1.docx]

***Supplementary Material***

Supplement A Sensitivity of the Diabetes definition

Supplement B Anthropometric, Geriatric, and Laboratory Assessments

Supplement Figure 1: Overview of the Berlin Initiative Study (BIS) population. The flowchart shows the composition of the study population. The light gray section of the figure indicates the part of the BIS study that preceded this study. Frailty assessment was implemented at the 3rd follow-up (FU) of the BIS, defining the baseline visit for this study.

Supplement Table 1 Main characteristics of women by combined diabetes/frailty categories

Supplement Table 2 Main characteristics of men by combined diabetes/frailty categories

Supplement Table 3 Persons at risk, cumulative event count for Figure 2a: Women

Supplement Table 4 Persons at risk, cumulative event count for Figure 2b: Men

Supplement C Main analyses for the total study population:

- Main characteristics of study population by combined diabetes/frailty categories
- Kaplan-Meier curves of the survival probabilities by combined diabetes/frailty categories of the total population
- Persons at risk, cumulative event count and median survival times for the total population
- Interaction between diabetes and frailty on the risk of mortality

Supplement D Strobe Statement

**Supplement A: Sensitivity of the Diabetes definition**

Of 296 individuals with diabetes 63 participants met the definition based only on an elevated HbA1c level without reporting diabetes medication. As the cut-off value of HbA1c ≥6.5% has been discussed in older adults and higher cut-off levels up to 8.0% have been proposed we further challenged our definition [1]. For this, we used the AOK claims data to verify our diabetes definition in those who were only eligible via the HbA1c level. Out of those 63 participants 57 had at least five International Classification of Diseases, Tenth Revision (ICD-10) codes for diabetes (ICD-10-code E11: 56 individuals and 1 individual ICD-10-code: E14) prior to study baseline supporting a diabetes type 2 diagnosis. Of the remaining 6 individuals 2 exceeded the threshold value of 7.5% for an increased risk leaving 4 participants that might have been misclassified do to our HbA1c threshold of 6.5%. We decided to leave those four individuals classified as having diabetes, since guidelines for diabetes type 2 proposed that in community-dwelling older adults the cut-off value off 6.5% still holds true [2-4].

In conclusion, 97.3% of the 296 individuals with diabetes were also assigned the ICD-10 code E11 for type 2 diabetes in the AOK data.

**Supplement B: Anthropometric, Geriatric, and Laboratory Assessments**

Anthropometric measures included calf circumference, hip and waist circumferences, height, and weight. Body Mass Index (BMI) was calculated by dividing weight in kilograms by the square of height in meters.

The geriatric assessments additionally to the modified frailty phenotype by Fried [5, 6] applied in the BIS include Timed Up and Go Test (mobility impairments assessment), Care Dependency (amount of time - at least 90 min per day over a period of at least 6 months - needed daily for substantial assistance in at least two activities of daily living in the personal hygiene, nutrition, and mobility categories, and, additionally, assistance with domestic tasks), Brief Pain Inventory (pain, pain intensity assessment and pain impairment) and Mental Health Inventory (MHI-5; psychological well-being assessment; short version of Mental Health Inventory). Questions about falls (fall and fall situation in the last 4 weeks), urinary incontinence (qualitative and quantitative assessment of urinary incontinence), and sleep (sleep satisfaction assessment) were developed as part of research on geriatric health at Charité – Universitätsmedizin Berlin [7].

Laboratory analyses included hematological parameters measured in EDTA whole blood (white blood cell count, red blood cell count, hemoglobin, hematocrit, platelet count, MCV, MCH, MCHC), serum biomarkers (creatinine, albumin, C-reactive protein, total cholesterol, triglycerides, HDL cholesterol, LDL cholesterol, calcium, phosphate, glycated hemoglobin [HbA1c], uric acid, urea, cystatin C, sodium), and urine measurements (albumin, creatinine).

**References**

1. American Diabetes, A., *Standards of Care in Diabetes-2023 Abridged for Primary Care Providers.* Clin Diabetes, 2022. **41**(1): p. 4–31.

2. Masuch, A., et al., *Preventing misdiagnosis of diabetes in the elderly: age-dependent HbA1c reference intervals derived from two population-based study cohorts.* BMC Endocr Disord, 2019. **19**(1): p. 20.

3. Bundesärztekammer (BÄK), Kassenärztliche Bundesvereinigung (KBV), and Arbeitsgemeinschaft der wissenschaftlichen Medizinischen Fachgesellschaften (AWMF). *Nationale VersorgungsLeitlinie Typ-2-Diabetes – Langfassung. Version 3.0.* 2023 03.02.2025]; Available from: [www.leitlinien.de/diabetes](file:///\\Charite.de\Centren\C01\IPH\BIS\Projects\Frailty\Frailty_diabetes\Manuskript\Frontiers%20in%20Endocrinology\www.leitlinien.de\diabetes).

4. Brockamp, C., et al., *Shared Decision Making, Diagnostic Evaluation, and Pharmacotherapy in Type 2 Diabetes.* Dtsch Arztebl Int, 2023. **120**(47): p. 804–810.

5. Fried, L.P., et al., *Frailty in older adults: evidence for a phenotype.* J Gerontol A Biol Sci Med Sci, 2001. **56**(3): p. M146–56.

6. Mielke, N., et al., *Gender differences in frailty transition and its prediction in community-dwelling old adults.* Sci Rep, 2022. **12**(1): p. 7341.

7. Schnitzer, S., et al., *Risk Profiles for Care Dependency: Cross-Sectional Findings of a Population-Based Cohort Study in Germany.* J Aging Health, 2020. **32**(5-6): p. 352–360.


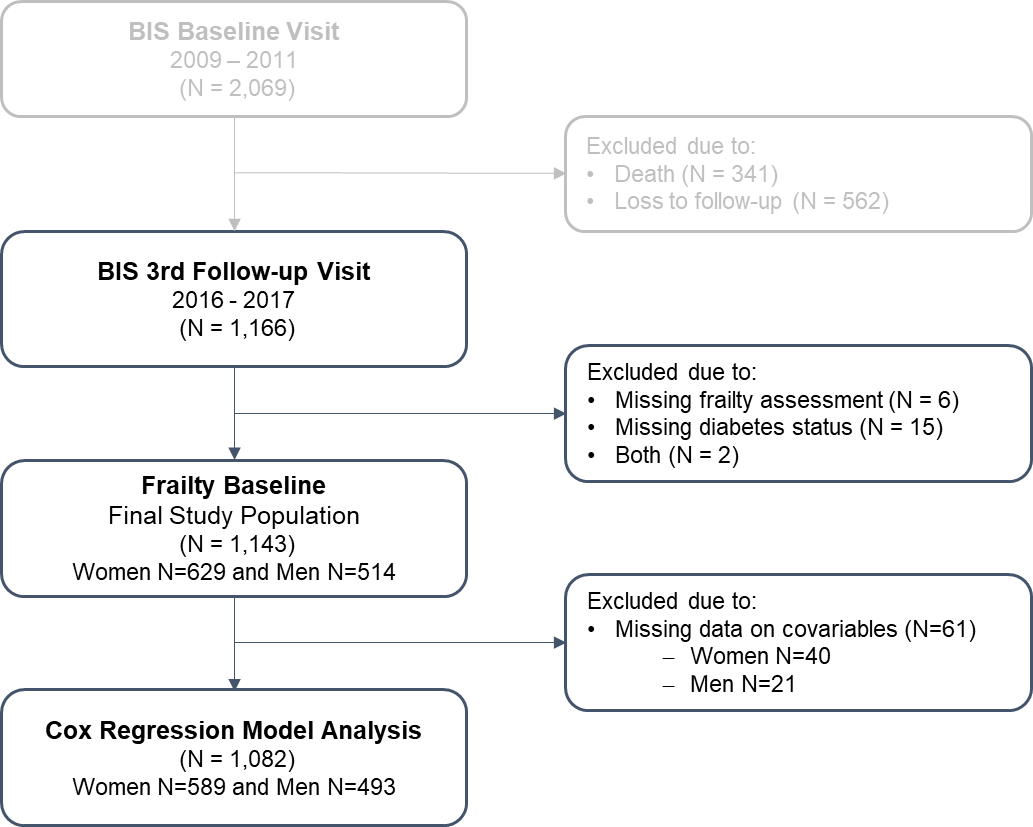


Supplement Figure 1: Overview of the Berlin Initiative Study (BIS) population. The flowchart shows the composition of the study population. The light gray section of the figure indicates the part of the BIS study that preceded this study. Frailty assessment was implemented at the 3rd follow-up (FU) of the BIS, defining the baseline visit for this study.

**Supplement Table 1: Main characteristics of women by combined diabetes/frailty categories**

|  |  | **Combination Diabetes/Frailty Categories** | | | |
| --- | --- | --- | --- | --- | --- |
|  | **Total (N=629)** | **No diabetes /Non-Frail (N=318)** | **Diabetes /Non-Frail (N=78)** | **No diabetes /Frail (N=164)** | **Diabetes /Frail (N=69)** |
| **Age (Years), mean (SD)** | | | | | |
| Mean (SD) | 84.0 (5.6) | 82.4 (4.8) | 82.6 (4.6) | 87.1 (5.9) | 86.1 (5.3) |
| **CASMIN, N (%)** | | | | | |
| Low | 412 (66) | 199 (63) | 58 (74) | 111 (68) | 44 (64) |
| Intermediate | 147 (23) | 75 (24) | 15 (19) | 37 (23) | 20 (29) |
| High | 69 (11) | 43 (14) | 5 (6) | 16 (10) | 5 (7) |
| Missing | 1 (0.2) | 1 (0.3) | 0 (0) | 0 (0) | 0 (0) |
| **Partner Status, N (%)** | | | | | |
| No | 418 (66) | 191 (60) | 48 (62) | 126 (77) | 53 (77) |
| Yes | 208 (33) | 126 (40) | 30 (38) | 37 (23) | 15 (22) |
| Missing | 3 (0.5) | 1 (0.3) | 0 (0) | 1 (0.6) | 1 (1.4) |
| **Body Mass Index (kg/m^2^), N (%)** | | | | | |
| < 22 | 78 (12) | 47 (15) | 5 (6) | 24 (15) | 2 (3) |
| ≥ 22 - < 30 | 392 (62) | 212 (67) | 48 (62) | 99 (60) | 33 (48) |
| ≥ 30 | 154 (24) | 59 (19) | 25 (32) | 38 (23) | 32 (46) |
| Missing | 5 (0.8) | 0 (0) | 0 (0) | 3 (1.8) | 2 (2.9) |
| **Smoking, N (%)** | | | | | |
| Never | 453 (72) | 229 (72) | 60 (77) | 118 (72) | 46 (67) |
| Ever | 174 (28) | 89 (28) | 18 (23) | 44 (27) | 23 (33) |
| Missing | 2 (0.3) | 0 (0) | 0 (0) | 2 (1.2) | 0 (0) |
| **Total Cholesterol (mg/dl)** | | | | | |
| Mean (SD) | 220 (48) | 230 (48) | 200 (47) | 210 (47) | 210 (45) |
| Missing (%) | 15 (2.4) | 0 (0) | 3 (3.8) | 10 (6.1) | 2 (2.9) |
| **C-reactive Protein (mg/l)** | | | | | |
| Median (IQR) | 1.7 (0.9, 3.6) | 1.3 (0.8, 2.5) | 2.5 (1.0, 5.0) | 1.9 (1.0, 4.5) | 3.3 (1.4, 7.8) |
| Missing (%) | 18 (2.9) | 2 (0.6) | 3 (3.8) | 11 (6.7) | 2 (2.9) |
| **Charlson Comorbidity Index, median (IQR)** | | | | | |
|  | 5 (3, 7) | 4 (2, 6) | 6 (4, 8) | 7 (4, 8) | 9 (6, 11) |
| Missing | 9 (1.4) | 6 (1.9) | 0 (0) | 3 (1.8) | 0 (0) |
| **Treated Arterial Hypertension, N (%)** | | | | | |
| No | 100 (16) | 63 (20) | 5 (6) | 28 (17) | 4 (6) |
| Yes | 528 (84) | 255 (80) | 73 (94) | 136 (83) | 64 (93) |
| Missing | 1 (0.2) | 0 (0) | 0 (0) | 0 (0) | 1 (1.4) |
| **Cardiovascular Disease, N (%)** | | | | | |
| No | 201 (32) | 134 (42) | 25 (32) | 38 (23) | 4 (6) |
| Yes | 422 (67) | 180 (57) | 53 (68) | 126 (77) | 63 (91) |
| Missing | 6 (1.0) | 4 (1.3) | 0 (0) | 0 (0) | 2 (2.9) |
| **Chronic Kidney Disease, N (%)** | | | | | |
| No | 120 (19) | 91 (29) | 9 (12) | 16 (10) | 4 (6) |
| Yes | 485 (77) | 221 (69) | 66 (85) | 136 (83) | 62 (90) |
| Missing | 24 (3.8) | 6 (1.9) | 3 (3.8) | 12 (7.3) | 3 (4.3) |
| **Age of Diabetes Onset, N (%)** | | | | | |
| <70 years | 50 (8) | na | 25 (32) | na | 25 (36) |
| >=70 years | 90 (14) | na | 49 (63) | na | 41 (59) |
| Unknown | 7 (1) | na | 4 (5) | na | 3 (4) |

CASMIN Comparative Analysis of Social Mobility in Industrial Nations, BMI body mass index, CKD chronic kidney disease defined as either glomerular filtration rate of <= 60 ml/min per 1.73 m^2^ or ACR (albumin-creatinine ratio) >= 30 mg/g, hypertension defined as intake of any antihypertensive medication, cardiovascular disease (ever stroke, ever myocardial infarction, congestive heart failure or peripheral vascular disease), na not applicable

**Supplement Table 2: Main characteristics of men by combined diabetes/frailty categories**

|  |  | Combination Diabetes/Frailty Categories | | | |
| --- | --- | --- | --- | --- | --- |
|  | **Total (N=514)** | **No diabetes /Non-Frail (N=268)** | **Diabetes /Non-Frail (N=91)** | **No diabetes /Frail (N=97)** | **Diabetes /Frail (N=58)** |
| Age (Years), mean (SD) | | | | | |
| Mean (SD) | 84.3 (5.7) | 83.5 (5.0) | 83.0 (4.9) | 88.5 (5.9) | 86.7 (6.0) |
| CASMIN, N (%) | | | | | |
| Low | 265 (52) | 142 (53) | 49 (54) | 44 (45) | 30 (52) |
| Intermediate | 87 (17) | 43 (16) | 14 (15) | 20 (21) | 10 (17) |
| High | 158 (31) | 83 (31) | 26 (29) | 32 (33) | 17 (29) |
| Missing | 4 (0.8) | 0 (0) | 2 (2.2) | 1 (1.0) | 1 (1.7) |
| Partner Status, N (%) | | | | | |
| No | 152 (30) | 70 (26) | 20 (22) | 38 (39) | 24 (41) |
| Yes | 361 (70) | 198 (74) | 71 (78) | 59 (61) | 33 (57) |
| Missing | 1 (0.2) | 0 (0) | 0 (0) | 0 (0) | 1 (1.7) |
| Body Mass Index (kg/m^2^), N (%) | | | | | |
| < 22 | 34 (7) | 17 (6) | 2 (2) | 12 (12) | 3 (5) |
| ≥ 22 - < 30 | 371 (72) | 208 (78) | 66 (73) | 68 (70) | 29 (50) |
| ≥ 30 | 101 (20) | 43 (16) | 23 (25) | 14 (14) | 21 (36) |
| Missing | 8 (1.6) | 0 (0) | 0 (0) | 3 (3.1) | 5 (8.6) |
| Smoking, N (%) | | | | | |
| Never | 162 (32) | 90 (34) | 23 (25) | 29 (30) | 20 (34) |
| Ever | 351 (68) | 178 (66) | 68 (75) | 68 (70) | 37 (64) |
| Missing | 1 (0.2) | 0 (0) | 0 (0) | 0 (0) | 1 (1.7) |
| Total Cholesterol (mg/dl) | | | | | |
| Mean (SD) | 190 (41) | 190 (43) | 180 (35) | 180 (40) | 170 (37) |
| Missing (%) | 5 (1.0) | 0 (0) | 0 (0) | 0 (0) | 5 (8.6) |
| C-reactive Protein (mg/l) | | | | | |
| Median (IQR) | 1.6 (0.8, 3.5) | 1.3 (0.7, 2.5) | 1.7 (0.8, 3.4) | 2.6 (1.1, 5.8) | 3.1 (1.2, 7.3) |
| Missing (%) | 5 (1.0) | 0 (0) | 0 (0) | 0 (0) | 5 (8.6) |
| Charlson Comorbidity Index, median (IQR) | | | | | |
|  | 6 (4, 9) | 5 (3, 7) | 7 (5, 9) | 7 (5, 10) | 9 (6, 10) |
| Missing | 6 (1.2) | 5 (1.9) | 0 (0) | 0 (0) | 1 (1.7) |
| Treated Arterial Hypertension, N (%) | | | | | |
| No | 95 (18) | 64 (24) | 12 (13) | 14 (14) | 5 (9) |
| Yes | 419 (82) | 204 (76) | 79 (87) | 83 (86) | 53 (91) |
| Cardiovascular Disease, N (%) | | | | | |
| No | 114 (22) | 91 (34) | 15 (16) | 3 (3) | 5 (9) |
| Yes | 395 (77) | 173 (65) | 76 (84) | 93 (96) | 53 (91) |
| Missing | 5 (1.0) | 4 (1.5) | 0 (0) | 1 (1.0) | 0 (0) |
| Chronic Kidney Disease, N (%) | | | | | |
| No | 103 (20) | 73 (27) | 16 (18) | 9 (9) | 5 (9) |
| Yes | 405 (79) | 194 (72) | 75 (82) | 88 (91) | 48 (83) |
| Missing | 6 (1.2) | 1 (0.4) | 0 (0) | 0 (0) | 5 (8.6) |
| Age of Diabetes Onset, N (%) | | | | | |
| <70 years | 66 (13) | na | 39 (43) | na | 27 (47) |
| >=70 years | 75 (15) | na | 46 (51) | na | 29 (50) |
| Unknown | 8 (2) | na | 6 (7) | na | 2 (3) |

CASMIN Comparative Analysis of Social Mobility in Industrial Nations, BMI body mass index, CKD chronic kidney disease defined as either glomerular filtration rate of <= 60 ml/min per 1.73 m^2^ or ACR (albumin-creatinine ratio) >= 30 mg/g, hypertension defined as intake of any antihypertensive medication, cardiovascular disease (ever stroke, ever myocardial infarction, congestive heart failure or peripheral vascular disease), na not applicable

**Supplement Table 3: Persons at risk, cumulative event count for Figure 2a: Women**

|  | Time, months | | | | | | | | |
| --- | --- | --- | --- | --- | --- | --- | --- | --- | --- |
|  | 0 | 10 | 20 | 30 | 40 | 50 | 60 | 70 | 80 |
| **Persons at risk** |  |  |  |  |  |  |  |  |  |
| No diabetes /Non-Frail | 305 | 302 | 297 | 289 | 283 | 276 | 263 | 233 | 102 |
| Diabetes /Non-Frail | 74 | 72 | 71 | 68 | 62 | 59 | 55 | 51 | 21 |
| No diabetes /Frail | 148 | 142 | 133 | 125 | 116 | 98 | 91 | 62 | 32 |
| Diabetes /Frail | 62 | 53 | 48 | 45 | 42 | 41 | 32 | 17 | 7 |
|  |  |  |  |  |  |  |  |  |  |
| **Cumulative event count** |  |  |  |  |  |  |  |  |  |
| No diabetes /Non-Frail | 0 | 3 | 8 | 16 | 22 | 29 | 42 | 72 | 203 |
| Diabetes /Non-Frail | 0 | 2 | 3 | 6 | 12 | 15 | 19 | 23 | 53 |
| No diabetes /Frail | 0 | 6 | 15 | 23 | 32 | 50 | 57 | 86 | 116 |
| Diabetes /Frail | 0 | 9 | 14 | 17 | 20 | 21 | 30 | 45 | 55 |
| **Median survival times, in Months (IQR)** | | | |  |  |  |  |  |  |
| No diabetes /Non-Frail | NA |  |  |  |  |  |  |  |  |
| Diabetes /Non-Frail | NA |  |  |  |  |  |  |  |  |
| No diabetes /Frail | 69.4 (62.6-NA) | | |  |  |  |  |  |  |
| Diabetes /Frail | 60.6 (56.2-71.9) | | |  |  |  |  |  |  |

NA: Not Available

**Supplement Table 4: Persons at risk, cumulative event count for Figure 2b: Men**

|  | Time, months | | | | | | | | |
| --- | --- | --- | --- | --- | --- | --- | --- | --- | --- |
|  | 0 | 10 | 20 | 30 | 40 | 50 | 60 | 70 | 80 |
| **Persons at risk** |  |  |  |  |  |  |  |  |  |
| No diabetes /Non-Frail | 263 | 260 | 252 | 242 | 226 | 205 | 193 | 171 | 71 |
| Diabetes /Non-Frail | 89 | 88 | 86 | 83 | 74 | 65 | 55 | 49 | 20 |
| No diabetes /Frail | 92 | 81 | 65 | 56 | 46 | 36 | 28 | 22 | 9 |
| Diabetes /Frail | 49 | 45 | 38 | 30 | 28 | 21 | 16 | 10 | 3 |
|  |  |  |  |  |  |  |  |  |  |
| **Cumulative event count** |  |  |  |  |  |  |  |  |  |
| No diabetes /Non-Frail | 0 | 3 | 11 | 21 | 37 | 58 | 70 | 92 | 192 |
| Diabetes /Non-Frail | 0 | 1 | 3 | 6 | 15 | 24 | 34 | 40 | 69 |
| No diabetes /Frail | 0 | 11 | 27 | 36 | 46 | 56 | 64 | 70 | 83 |
| Diabetes /Frail | 0 | 4 | 11 | 19 | 21 | 28 | 33 | 39 | 46 |
| **Median survival times, in Months (IQR)** | | | |  |  |  |  |  |  |
| No diabetes /Non-Frail | NA |  |  |  |  |  |  |  |  |
| Diabetes /Non-Frail | 78.3 (63.8-NA) | | |  |  |  |  |  |  |
| No diabetes /Frail | 39.9 (31.8-50.3) | | |  |  |  |  |  |  |
| Diabetes /Frail | 42.0 (30.0-56.9) | | |  |  |  |  |  |  |

NA: Not Available

**Supplement B – Main analyses for the total study population**

**Main characteristics of study population by combined diabetes/frailty categories**

|  |  | **Combination Diabetes/Frailty Categories** | | | |
| --- | --- | --- | --- | --- | --- |
|  | **Total (N=1143)** | **No diabetes /Non-Frail (N=586)** | **Diabetes /Non-Frail (N=169)** | **No diabetes /Frail (N=261)** | **Diabetes /Frail (N=127)** |
| **Age (Years), mean (SD)** | 84.3 (5.6) | 82.9 (4.9) | 82.8 (4.8) | 87.6 (5.9) | 86.4 (5.6) |
| **sex, n (%)**  **Women** | 629 (55) | 318 (54) | 78 (46) | 164 (63) | 69 (54) |
| **CASMIN, N (%)** | | | | | |
| Low | 677 (59) | 341 (58) | 107 (63) | 155 (59) | 74 (58) |
| Intermediate | 234 (20) | 118 (20) | 29 (17) | 57 (22) | 30 (24) |
| High | 227 (20) | 126 (22) | 31 (18) | 48 (18) | 22 (17) |
| Missing | 5 (0.4) | 1 (0.2) | 2 (1.2) | 1 (0.4) | 1 (0.8) |
| **Partner Status, N (%)** | | | | | |
| Yes | 569 (50) | 324 (55) | 101 (60) | 96 (37) | 48 (38) |
| Missing | 4 (0.4) | 1 (0.2) | 0 (0) | 1 (0.4) | 2 (1.6) |
| **Body Mass Index (kg/m^2^), N (%)** | | | | | |
| < 22 | 112 (10) | 64 (11) | 7 (4) | 36 (14) | 5 (4) |
| ≥ 22 - < 30 | 763 (67) | 420 (72) | 114 (67) | 167 (64) | 62 (49) |
| ≥ 30 | 255 (22) | 102 (17) | 48 (28) | 52 (20) | 53 (42) |
| Missing | 13 (1.1) | 0 (0) | 0 (0) | 6 (2.3) | 7 (5.5) |
| **Smoking, N (%)** | | | | | |
| Never | 615 (54) | 319 (54) | 83 (49) | 147 (56) | 66 (52) |
| Ever | 525 (46) | 267 (46) | 86 (51) | 112 (43) | 60 (47) |
| Missing | 3 (0.3) | 0 (0) | 0 (0) | 2 (0.8) | 1 (0.8) |
| **Total Cholesterol (mg/dl)** | | | | | |
| Mean (SD) | 200 (48) | 210 (49) | 190 (43) | 200 (47) | 190 (46) |
| Missing (%) | 20 (1.7) | 0 (0) | 3 (1.8) | 10 (3.8) | 7 (5.5) |
| **C-reactive Protein (mg/l)** | | | | | |
| Median (IQR) | 1.7 (0.9, 3.6) | 1.3 (0.7, 2.5) | 1.9 (0.9, 4.0) | 2.2 (1.0, 5.4) | 3.3 (1.2, 7.7) |
| Missing (%) |  |  |  |  |  |
| **Charlson Comorbidity Index** | | | | | |
| Median (IQR) | 6 (3, 8) | 4 (3, 6) | 6 (4, 9) | 7 (4, 9) | 9 (6, 11) |
| Missing |  |  |  |  |  |
| **Treated Arterial Hypertension, N (%)** | | | | | |
| Yes | 947 (83) | 459 (78) | 152 (90) | 219 (84) | 117 (92) |
| Missing | 1 (0.1) | 0 (0) | 0 (0) | 0 (0) | 1 (0.8) |
| **Cardiovascular Disease, N (%)** | | | | | |
| Yes | 817 (71) | 353 (60) | 129 (76) | 219 (84) | 116 (91) |
| Missing | 11 (1.0) | 8 (1.4) | 0 (0) | 1 (0.4) | 2 (1.6) |
| **Chronic Kidney Disease, N (%)** | | | | | |
| Yes | 890 (78) | 415 (71) | 141 (83) | 224 (86) | 110 (87) |
| Missing | 30 (2.6) | 7 (1.2) | 3 (1.8) | 12 (4.6) | 8 (6.3) |
| **Age of Diabetes Onset, N (%)** | | | | | |
| <70 years | 116 (10) | na | 64 (38) | na | 52 (41) |
| >=70 years | 165 (14) | na | 95 (56) | na | 70 (55) |
| Unknown | 15 (1) | na | 10 (6) | na | 5 (4) |

CASMIN Comparative Analysis of Social Mobility in Industrial Nations, BMI body mass index, CKD chronic kidney disease defined as either glomerular filtration rate of <= 60 ml/min per 1.73 m^2^ or ACR (albumin-creatinine ratio) >= 30 mg/g, hypertension defined as intake of any antihypertensive medication, cardiovascular disease (ever stroke, ever myocardial infarction, congestive heart failure or peripheral vascular disease), na not applicable


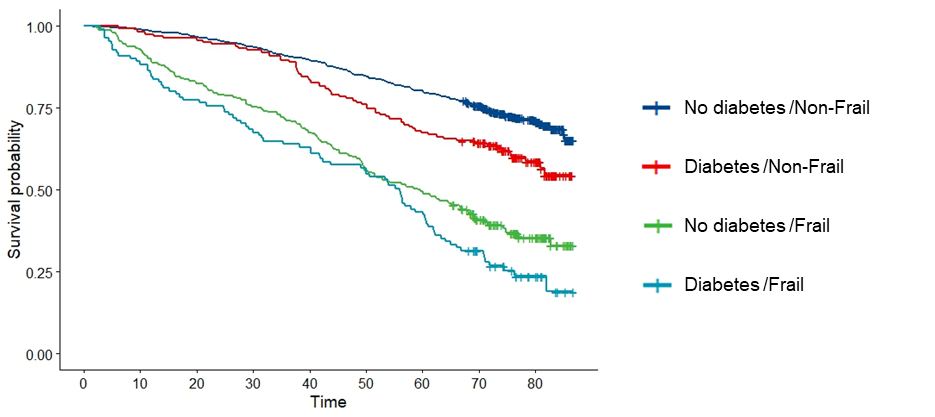


**Kaplan-Meier curves of the survival probabilities over time (months) by combined diabetes/frailty categories of the total population**

**Persons at risk, cumulative event count and median survival times for the total population**

|  | Time, months | | | | | | | | |
| --- | --- | --- | --- | --- | --- | --- | --- | --- | --- |
|  | 0 | 10 | 20 | 30 | 40 | 50 | 60 | 70 | 80 |
| **Persons at risk** |  |  |  |  |  |  |  |  |  |
| No diabetes /Non-Frail | 568 | 562 | 549 | 531 | 509 | 481 | 456 | 404 | 173 |
| Diabetes /Non-Frail | 163 | 160 | 157 | 151 | 136 | 124 | 110 | 100 | 41 |
| No diabetes /Frail | 240 | 223 | 198 | 181 | 162 | 134 | 119 | 84 | 41 |
| Diabetes /Frail | 111 | 98 | 86 | 75 | 70 | 62 | 48 | 27 | 10 |
|  |  |  |  |  |  |  |  |  |  |
| **Cumulative event count** |  |  |  |  |  |  |  |  |  |
| No diabetes /Non-Frail | 0 | 6 | 19 | 37 | 59 | 87 | 112 | 164 | 395 |
| Diabetes /Non-Frail | 0 | 3 | 6 | 12 | 27 | 39 | 53 | 63 | 122 |
| No diabetes /Frail | 0 | 17 | 42 | 59 | 78 | 106 | 121 | 156 | 199 |
| Diabetes /Frail | 0 | 13 | 25 | 36 | 41 | 49 | 63 | 84 | 101 |
| **Median survival times, in Months (IQR)** | | | |  |  |  |  |  |  |
| No diabetes /Non-Frail | NA |  |  |  |  |  |  |  |  |
| Diabetes /Non-Frail | NA |  |  |  |  |  |  |  |  |
| No diabetes /Frail | 59.4 (49.8-68.2) | | |  |  |  |  |  |  |
| Diabetes /Frail | 56.0 (43.7-60.7) | | |  |  |  |  |  |  |

NA: Not Available

**Interaction between diabetes and frailty on the risk of mortality**

|  |  | **Number of participants** | **Deaths** | **Person years** | **HR (95% CI)** |
| --- | --- | --- | --- | --- | --- |
| **Combined categories** | |  |  |  |  |
| No diabetes / Non-Frail | | 568 | 162 | 3258 | Reference |
| Diabetes /Non-Frail | | 163 | 66 | 875 | 1.42 (1.06, 1.90) |
| No diabetes / Frail | | 240 | 151 | 1049 | 2.09 (1.63, 2.67) |
| Diabetes /Frail |  | 111 | 83 | 440 | 3.39 (2.53, 4.55) |
| **Stratified by Frailty** | |  |  |  |  |
| Non-Frail | No Diabetes | 568 | 162 | 3258 | Reference |
|  | Diabetes | 163 | 66 | 875 | 1.42 (1.06, 1.90) |
| Frail | No Diabetes | 240 | 151 | 1049 | Reference |
|  | Diabetes | 111 | 83 | 440 | 1.62 (1.22, 2.16) |
| **Stratified by diabetes** | |  |  |  |  |
| No Diabetes | Non-Frail | 568 | 162 | 3258 | Reference |
|  | Frail | 240 | 151 | 1049 | 2.09 (1.63, 2.67) |
| Diabetes | Non-Frail | 163 | 66 | 875 | Reference |
|  | Frail | 111 | 83 | 440 | 2.39 (1.71, 3.34) |
|  |  |  |  |  |  |
| Ratio of HRs (95% CI) | |  |  |  | 1.14 (0.77, 1.71) |
|  |  |  |  |  |  |
| RERI (95% CI) |  |  |  |  | 0.88 (-0.03, 1.8) |

RERI: Relative excess risk due to interaction; HR: Hazard ratio; 95% CI: 95% Confidence interval

All HRs are adjusted for age, sex, CASMIN, Partner Status, BMI, smoking, Total cholesterol, CRP, Hypertension, CVD and CKD

**Supplement C: Strobe Statement**

STROBE Statement—Checklist of items that should be included in reports of ***cohort studies***

|  | | **Item No** | **Recommendation** | **Page No** |
| --- | --- | --- | --- | --- |
| **Title and abstract** | | 1 | (*a*) Indicate the study’s design with a commonly used term in the title or the abstract | Abstract |
|  |  |  | (*b*) Provide in the abstract an informative and balanced summary of what was done and what was found | Abstract |
| **Introduction** | | | | |
| Background/rationale | | 2 | Explain the scientific background and rationale for the investigation being reported | Introduction |
| Objectives | | 3 | State specific objectives, including any prespecified hypotheses | Introduction, last paragraph |
| **Methods** | | | | |
| Study design | | 4 | Present key elements of study design early in the paper | Methods – Study population |
| Setting | | 5 | Describe the setting, locations, and relevant dates, including periods of recruitment, exposure, follow-up, and data collection | Methods – Study population |
| Participants | | 6 | (*a*) Give the eligibility criteria, and the sources and methods of selection of participants. Describe methods of follow-up | Methods – Study population |
|  |  |  | (*b*) For matched studies, give matching criteria and number of exposed and unexposed | Not applicable |
| Variables | | 7 | Clearly define all outcomes, exposures, predictors, potential confounders, and effect modifiers. Give diagnostic criteria, if applicable | Methods - Exposures: Diabetes and Frailty, Outcome: all-cause mortality and Covariable assessment |
| Data sources/ measurement | | 8* | For each variable of interest, give sources of data and details of methods of assessment (measurement). Describe comparability of assessment methods if there is more than one group | Methods - Covariable assessment |
| Bias | | 9 | Describe any efforts to address potential sources of bias |  |
| Study size | | 10 | Explain how the study size was arrived at | Methods – Study population |
| Quantitative variables | | 11 | Explain how quantitative variables were handled in the analyses. If applicable, describe which groupings were chosen and why | Methods –Statistical  analyses |
| Statistical methods | | 12 | (*a*) Describe all statistical methods, including those used to control for confounding | Methods –Statistical  analyses |
|  |  |  | (*b*) Describe any methods used to examine subgroups and interactions |  |
|  |  |  | (*c*) Explain how missing data were addressed |  |
|  |  |  | (*d*) If applicable, explain how loss to follow-up was addressed |  |
|  |  |  | (*e*) Describe any sensitivity analyses |  |
| **Results** | | | |  |
| Participants | | 13* | (a) Report numbers of individuals at each stage of study—eg numbers potentially eligible, examined for eligibility, confirmed eligible, included in the study, completing follow-up, and analysed | Supplement Figure 1 |
|  |  |  | (b) Give reasons for non-participation at each stage |  |
|  |  |  | (c) Consider use of a flow diagram |  |
| Descriptive data | | 14* | (a) Give characteristics of study participants (eg demographic, clinical, social) and information on exposures and potential confounders | Results – Table 1 |
|  |  |  | (b) Indicate number of participants with missing data for each variable of interest |  |
|  |  |  | (c) Summarise follow-up time (eg, average and total amount) |  |
| Outcome data | | 15* | Report numbers of outcome events or summary measures over time | Results – Polypharmacy and incident frailty |
| Main results | 16 | (*a*) Give unadjusted estimates and, if applicable, confounder-adjusted estimates and their precision (eg, 95% confidence interval). Make clear which confounders were adjusted for and why they were included | | Table 2 and 3 |
|  |  | (*b*) Report category boundaries when continuous variables were categorized | |  |
|  |  | (*c*) If relevant, consider translating estimates of relative risk into absolute risk for a meaningful time period | |  |
| Other analyses | 17 | Report other analyses done—eg analyses of subgroups and interactions, and sensitivity analyses | | Table 4 |
| **Discussion** | | | | |
| Key results | 18 | Summarise key results with reference to study objectives | | Discussion – 1st  paragraph |
| Limitations | 19 | Discuss limitations of the study, taking into account sources of potential bias or imprecision. Discuss both direction and magnitude of any potential bias | | Discussion – before  last paragraph |
| Interpretation | 20 | Give a cautious overall interpretation of results considering objectives, limitations, multiplicity of analyses, results from similar studies, and other relevant evidence | | Discussion |
| Generalisability | 21 | Discuss the generalisability (external validity) of the study results | | Discussion |
| **Other information** | | | | |
| Funding | 22 | Give the source of funding and the role of the funders for the present study and, if applicable, for the original study on which the present article is based | | Funding paragraph |

*Give information separately for exposed and unexposed groups.

**Note:** An Explanation and Elaboration article discusses each checklist item and gives methodological background and published examples of transparent reporting. The STROBE checklist is best used in conjunction with this article (freely available on the Web sites of PLoS Medicine at http://www.plosmedicine.org/, Annals of Internal Medicine at http://www.annals.org/, and Epidemiology at http://www.epidem.com/). Information on the STROBE Initiative is available at http://www.strobe-statement.org.
